# A library of synthetic transcription activator-like effector-activated promoters for coordinated orthogonal gene expression in plants

**DOI:** 10.1111/tpj.12843

**Published:** 2015-04-29

**Authors:** Kathleen Brückner, Petra Schäfer, Ernst Weber, Ramona Grützner, Sylvestre Marillonnet, Alain Tissier

**Affiliations:** 1 Department of Cell and Metabolic Biology, Leibniz Institute of Plant BiochemistryWeinberg 3, 06120, Halle (Saale), Germany; 2 Icon Genetics GmbHWeinbergweg 22, 06120, Halle (Saale), Germany; Bayer HealthCare, Protein Engineering and AssaysNattermannallee 1, 50829, Cologne, Germany

**Keywords:** synthetic promoters, transcription activator-like effectors, orthogonality, metabolic engineering, transient assays, *Nicotiana benthamiana*, terpenoid, synthetic biology, plants, technical advance

## Abstract

A library of synthetic promoters containing the binding site of a single designer transcription activator-like effector (dTALE) was constructed. The promoters contain a constant sequence, consisting of an 18-base long dTALE-binding site and a TATA box, flanked by degenerate sequences of 49 bases downstream and 19 bases upstream. Forty-three of these promoters were sequenced and tested in transient assays in *Nicotiana benthamiana* using a GUS reporter gene. The strength of expression of the promoters ranged from around 5% to almost 100% of the viral 35S promoter activity. We then demonstrated the utility of these promoters for metabolic engineering by transiently expressing three genes for the production of a plant diterpenoid in *N. benthamiana*. The simplicity of the promoter structure shows great promise for the development of genetic circuits, with wide potential applications in plant synthetic biology and metabolic engineering.

## Introduction

One of the goals of synthetic biology is to establish artificial transcriptional networks for the expression of novel signalling or metabolic pathways. While it has been successfully done in unicellular microorganisms (Brophy and Voigt, 2014), the development of such transcriptional networks in higher eukaryotes, in particular in higher plants, presents additional difficulties due to the lack of sufficient available data, multicellularity and chromatin structure. One prerequisite for the construction of such networks is the availability of a range of transcriptional promoters of varying strengths that can be activated by orthogonal transcription factors (TFs). Since such promoters are not available in nature, they have to be designed, synthesized, assembled in functional transcription units and tested.

Several approaches have been adopted for promoter engineering, including random mutagenesis, saturation mutagenesis of poorly conserved regions and the engineering of hybrid promoters (Blazeck and Alper, 2013). Expression levels achieved so far have covered a range over one to three orders of magnitude (Blazeck and Alper, 2013), indicating that such sets of promoters are adequate to finely tune the expression of individual genes for engineering of pathways. An additional desirable property of synthetic promoters is their activation by orthogonal TFs. This is particularly relevant for multicellular organisms such as higher plants, for example if the aim is to express a biosynthesis pathway exclusively in one tissue or under specific conditions such as biotic or abiotic stresses. Most promoter libraries described so far in yeast are based on upstream activating sequences (UAS) of endogenous TFs (Blazeck and Alper, 2013). If the TF on which the design of these synthetic promoters is based is expressed constitutively, or only in specific tissues or conditions, this will considerably restrict their potential use. For constitutive TF, this will prevent activation in specific tissues; conversely, for tissue-specific TFs this will restrict the use of the promoters to a specific tissue, preventing their general application for other tissues or conditions. A set of promoters whose expression can be induced by an orthogonal TF would circumvent these problems. In this case, the localization and pattern of expression is determined by the orthogonal TF, which itself can be put under the control of a tissue-specific promoter, for example.

The recently discovered transcription activator-like effectors (TALEs) provide the perfect raw material for developing such a tool (Boch *et al*., 2009; Boch and Bonas, 2010). The modular structure of their DNA-binding site allows the design of truly orthogonal TFs by selecting a DNA sequence that does not occur in the genome, thus minimizing off-target gene activation. Since their discovery, TALEs have been used both for transcription activation and genome editing, the latter by fusing them to nucleases (de Lange *et al*., 2014). While tools for the construction of custom-designed TALEs are available (Weber *et al*., 2011), there are, to the best of our knowledge, no described resources for libraries of synthetic promoters that can be activated by custom-designed TALEs. Such a resource would not only allow the coordinated expression of metabolic pathway genes in specific cell types or under given conditions, but would also provide the building blocks to construct artificial transcriptional networks by introducing positive or negative feedback loops, or transcriptional cascades. To fill this gap, and motivated by our interest in plant metabolic engineering, we undertook the design and construction of libraries of synthetic TALE-activated promoters (STAPs), which we describe here. These were assembled in Golden Gate-compatible vectors to facilitate their downstream use (Engler *et al*., 2014). These STAPs were first evaluated with reporter genes (*GUS* and *GFP*) in transient assays in *Nicotiana benthamiana*, then, as proof of principle, tested in a metabolic engineering set-up for the production of a plant diterpene.

## Results

### Design and construction of a library of synthetic TALE-activated promoters

**Figure 1:**
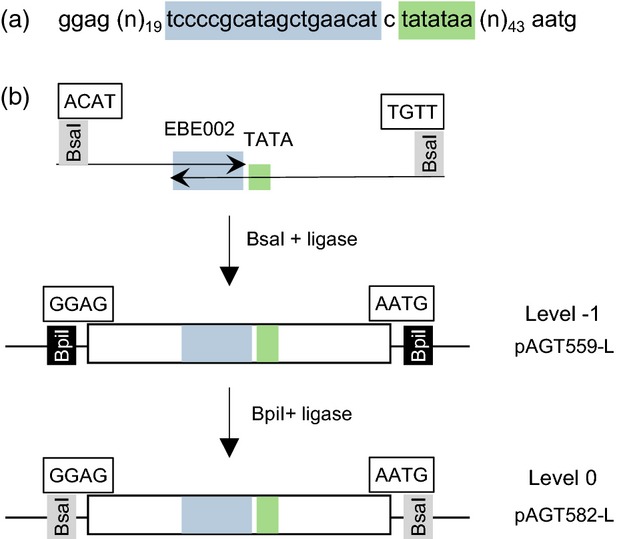
Design and cloning procedure of the synthetic transcription activator-like effector-activated promoters (STAPs).(a) general sequence of the STAPS. The constant designer transcription activator-like effector (dTALE)-binding site (EBE002) is highlighted in blue and the TATA box in green. The atg triplet at the end of the promoter corresponds to the translation start codon. The degenerate sequences upstream and downstream of the constant region are indicated by (n)_19_ and (n)_43_ respectively.(b) The STAPs cloning procedure. The library was synthesized using two degenerate oligonucleotides overlapping the constant region and cloned into a level –1 Golden Gate vector. Subsequent cloning into a level 0 vector allowed elimination of aberrant products (e.g. dimers) and false positives. The level 0 vectors can then be used for assembly into transcription units.

The structure of the STAPs is presented in Figure1(a). They contain an 18-base-long DNA-binding domain, named EBE002, flanked by a 19-base-long degenerate sequence immediately upstream, and a consensus TATA box downstream. The TATA box is followed by a 43-base-long degenerate sequence and the ATG start codon. This design is based on the observation that the DNA-binding sequence of TALEs from bacterial pathogens that activate genes *in planta* are typically located close to the transcription start site (less than 100 bp) and that insertion of a DNA-binding sequence at position –55 or –40 was sufficient to confer TALE-mediated inducibility (Kay *et al*., 2009). The library was cloned using the MoClo system based on Golden Gate cloning (Werner *et al*., 2012). The scheme for the construction of the STAPs library is presented in Figure1(b). The library was made in a level –1 vector of the MoClo system, yielding pAGT559-L, and transferred in a level 0 vector, yielding pAGT582-L. Level 0 vectors contain parts (typically promoter, cDNA, terminator) which are brought together in level 1 vectors to assemble functional transcription units. Transferring the library from level –1 to level 0 ensures that only fragments containing the correct overhangs are kept, thus limiting the number of false positives and increasing the efficiency of cloning in subsequent steps. Approximately 50 clones from the level 0 library were picked and checked for the presence of a fragment of the expected size. Forty-three clones were thus selected and sequenced (see Data S1 in Supporting Information). All promoter sequences contained the EBE002 DNA-binding site.

### Transient assays of the STAPs with a GUS reporter gene

**Figure 2:**
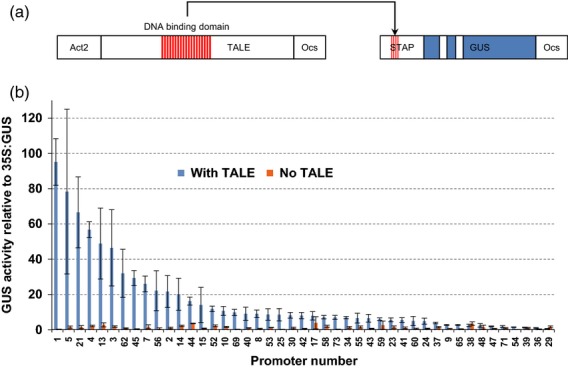
Testing the synthetic transcription activator-like effector-activated promoters (STAPs) in a transient assay with a *GUS* reporter gene.(a) Overview of the constructs used for the transient assay. The designer transcription activator-like effector (dTALE), which binds to the constant EBE002 sequence of the STAPs, is under the control of the constitutive and moderate *Act2* promoter. The STAPs control the expression of a *GUS* transgene with introns (represented by white bars within the blue coding sequence) to prevent expression in *Agrobacterium tumefaciens* (which is used for the infiltration of *Nicotiana benthamiana* leaves).(b) Distribution of the GUS activity values for the STAPs promoters with or without the dTALE, expressed in percentage of 35S:*GUS* expression.

Each STAP was cloned upstream of the *GUS* reporter gene and the ocs transcription terminator in a level 1 vector, which can also be used for *Agrobacterium tumefaciens* T-DNA transfer (Figure2a). The transcriptional activity of these promoters was first evaluated in transient assays in *Nicotiana benthamiana* by measuring GUS activity in protein extracts. The designer TALE (dTALE) is under control of the *Act2* promoter (Figure2a), which provides sufficient expression for the activation of transcription. To quantify the expression, leaves were infiltrated in triplicate with the STAP:*GUS* constructs, with or without the dTALE and with a 35S:*GFP* construct. The GFP fluorescence was measured and used as a normalization factor. The results (Figure2b) show a distribution of expression strengths ranging from less than 1% to over 90% of that of the 35S promoter. Since for some promoters there is significant residual background expression without the TALE, the useful range of expression in this transient assay is estimated to be between 5 and 90% of that of the 35S promoter. For most STAPs, however, no significant expression was detected in the absence of the TALE, indicating that the activity seen with the TALE does not stem from endogenous activators, establishing the orthogonality of the promoters.

### Transient assays of selected STAPs with GFP

**Figure 3:**
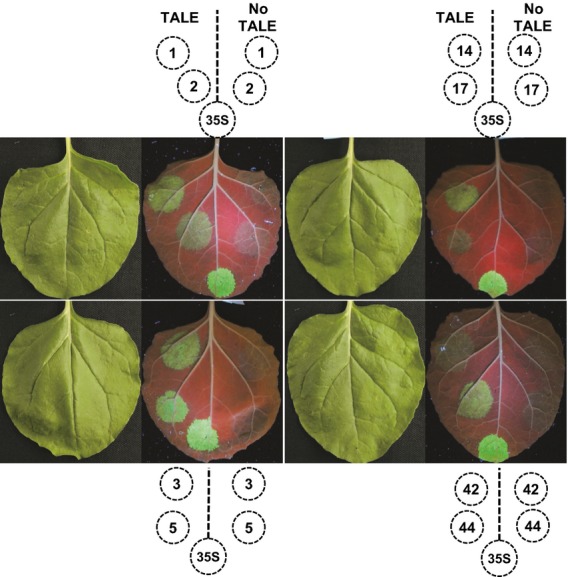
Testing selected synthetic transcription activator-like effector-activated promoters (STAPs) with GFP.Eight different STAPs displaying different levels of expression with the GUS reporter gene were tested with GFP. *Nicotiana benthamiana* leaves were infiltrated with *Agrobacterium tumefaciens* strains carrying different STAP:*GFP* constructs with (left side) or without (right side) the designer transcription activator-like effector (dTALE). Pictures of the leaves were taken in normal light (first and third columns) or under UV illumination (second and fourth columns) to visualize GFP.

To confirm these results with another reporter gene, a selection of eight STAPs with different expression strengths (STAPs numbers 1, 2, 3, 5, 14, 17, 42 and 44) were cloned in front of the GFP gene and transiently expressed in *N. benthamiana*. The results (Figure3) are consistent with those of the GUS assay, indicating that the strength of expression for GUS and GFP seems to be independent of the transcribed gene. No GFP signal could be seen in the absence of the TALE for any of the STAPs tested, confirming the high specificity of the synthetic promoters. The strongest promoters (numbers 1, 3 and 5) show a signal intensity approaching that of the 35S promoter, while the weaker promoters (numbers 17 and 42) still produce a detectable signal.

### Using STAPs for diterpene metabolic engineering in *N. benthamiana*

**Figure 4:**
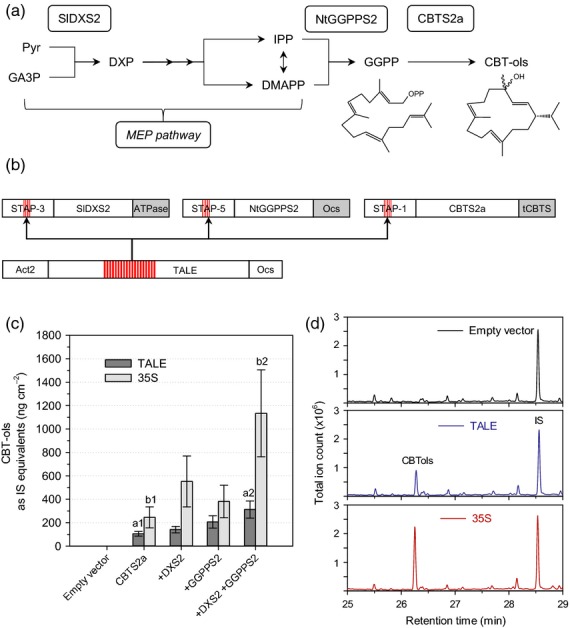
Using selected synthetic transcription activator-like effector-activated promoters (STAPs) for metabolic engineering of a plant diterpene.(a) overview of the pathway for cembratrienols (CBTols) including the methylerythritol-phosphate (MEP) pathway, highlighting the genes that were over-expressed in *Nicotiana benthamiana*. Pyr, pyruvate; GA3P, glyceraldehyde-3-phosphate; DXP, 1-deoxyxylulose 5-phosphate; IPP, isopentenyl diphosphate; DMAPP, dimethylallyl diphosphate; GGPP, geranylgeranyl diphosphate; SlDXS2, tomato 1-deoxyxylulose 5-phosphate synthase; NtGGPPS2, tobacco GGPP synthase; CBTS2a, tobacco cembratrienol synthase.(b) Overview of the constructs used for the overexpression of SlDXS2, NtGGPPS2 and CBTS2a using STAPs. The numbers of the STAPs correspond to the numbers in Figures2 and 3.(c) Comparison of transcription activator-like effector (TALE)-driven expression and p35S-driven expression in *N. benthamiana* leaves agro-infiltrated with *p19* and *CBTS2a*,*DXS2* and *GGPPS2* in different combinations. Two leaves in three individual plants were infiltrated for each construct combination (*n* = 6). Mean amounts of CBTol are expressed as equivalents of the internal standard (IS, sclareol) ± SE. Groups indicated by different letters (a1/a2, and b1/b2) differ significantly from each other regarding their CBTol values (*P* < 0.05; Student’s *t*-test and two-way anova). No CBTol was detected in leaves expressing the empty T-DNA vector as a control.

**Figure 5:**
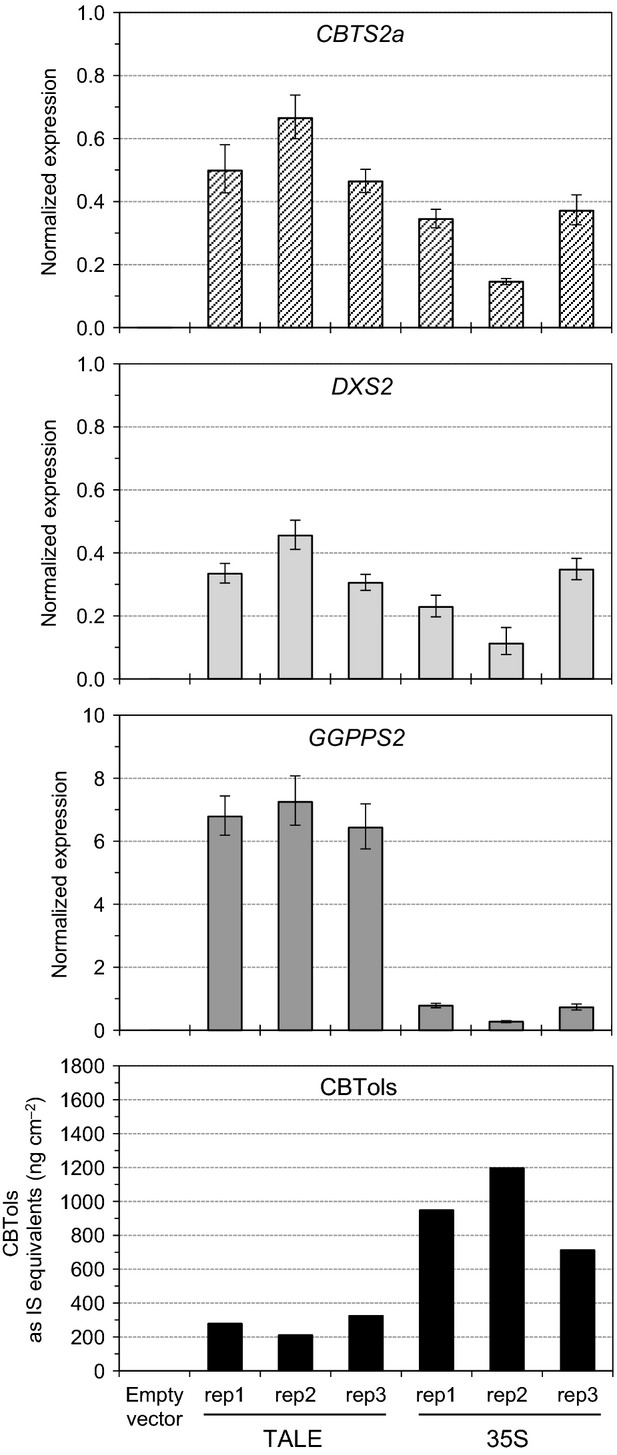
Transcription activator-like effector (TALE)- and 35S-driven expression of the *CBTS2a*,*DXS2* and *GGPPS2* genes in agro-infiltrated *Nicotiana benthamiana* leaves.Transcript levels were quantified by RT-PCR in three selected biological replicates from samples used in Figure4 (rep1-3) from agro-infiltrated leaves co-expressing *p19*,*CBTS2a*,*DXS2* and *GGPPS2* driven by synthetic transcription activator-like effector-activated promoters (TALE) or the 35S promoter (35S). Mean expression values of three technical replicates ± SD and the corresponding CBTol amounts for each sample are given.

We next sought to validate the utility of these promoters by using them for metabolic engineering of a plant diterpenoid. As output for our metabolic engineering assay we used cembratrienol synthase (CBTS2a) from tobacco (Wang and Wagner, 2003; Ennajdaoui *et al*., 2010). This diterpene synthase produces a mix of two stereoisomers (α and β) of the macrocyclic cembratrienol (CBTol) (Figure4). In plants, the terpenoid precursors isopentenyl diphosphate (IPP) and dimethylallyl diphosphate (DMAPP) are produced by two distinct pathways, the plastidic 2-*C*-methyl-d-erythritol-4-phosphate (MEP) pathway, and the cytosolic mevalonate (MEV) pathway (Vranova *et al*., 2012). Diterpenes are synthesized in the plastids and therefore predominantly use IPP and DMAPP from the MEP pathway. We have previously shown that co-expression of 1-deoxyxylulose 5-phosphate synthase (DXS), the first step of the MEP pathway, with a geranylgeranyl diphosphate synthase (GGPPS) leads to more than a doubling of diterpene production in *N. benthamiana* transient assays (Brückner and Tissier, 2013). These assays were carried out with the strong cauliflower mosaic virus 35S promoter controlling the expression of all the genes. The *DXS2* (tomato), *GGPPS2* (tobacco) and the *CBTS2a* genes were cloned downstream of some of the strongest STAPs characterized above and transiently expressed together with the dTALE. The results show that both with the 35S promoter and the STAPs, the amount of CBTol produced is significantly higher when all genes (*CBTS2a*,*GGPPS2* and *DXS2*) are co-expressed than with the other combinations (*CBTS2a* alone, *CBTS2a* + *DXS2* or *CBTS2a* + *GGPPS2*) (Figure4c), confirming previous results with the 35S promoter (Brückner and Tissier, 2013). However, the amount of CBTol produced with the the STAPs was around three-fold lower than with 35S. One explanation for this could be that gene expression is lower. To check this, we measured transcript levels by quantitative RT-PCR from the same samples used for quantification of CBTol. Surprisingly, the transcript levels were higher with the STAPs than with the 35S promoter (1.9 times for *CBTS2a*, 11.5 for *GGPPS2* and 1.5 for *DXS2*) (Figure5). Thus, the lower CBTol levels cannot be caused by reduced transcript levels of the genes. Plotting the gene transcript levels versus the amount of CBTol produced indicates a negative correlation, regardless of the promoters used (STAPs or 35S) (Figure S1). Thus, this inverse effect of gene expression on metabolite levels does not seem to be specific to STAPs.

These results show that STAPs can be used for metabolic engineering applications and can deliver expression levels which are comparable to those of the strongest known promoters.

## Discussion

We report here on the design, construction and testing of a library of synthetic promoters activated by a dTALE TF in plants. Based on a remarkably simple architecture consisting of the dTALE DNA-binding site, a TATA box and degenerate sequences flanking these constant regions, these STAPs exhibit properties that make them ideal tools for the development of complex transcriptional networks or for metabolic engineering applications. First, based on the *GUS* reporter gene, they exhibit a usable range of expression levels in transient assays that varies from 5 to 90% of that of the 35S promoter, which is one of the strongest plant promoters available. However, it is possible that, due to the high load of T-DNAs that are transferred, transient assays in *N. benthamiana* lead to background transcription activity. Whether this is still the case in stable transgenics remains to be seen. Large variations in expression levels of individual genes within a biosynthesis pathway have been observed in plants. For example, genes encoding the cytochrome P450 monooxygenases of the parthenolide pathway in feverfew (*Tanacetum parthenium*) can be expressed at levels several 100-fold higher than the gene encoding germacrene A synthase, which represents the first committed step of the pathway (Liu *et al*., 2014). Thus, the range of expression levels delivered by STAPs should prove particularly useful for tuning the expression of individual pathway genes in metabolic engineering projects. This can serve to optimize pathway flux but also to avoid excess production of certain enzymes, which may cause metabolic overload of the host cells. Second, in the absence of the dTALE, no or just background levels of expression were detected, demonstrating the orthogonal nature of the dTALE/STAP system.

We could also validate the utility of STAPs for metabolic engineering in a transient assay. We could confirm that co-expression of GGPPS2 and DXS2 with the diterpene synthase CBTS2a leads to increased levels of CBTol (Figure4c). However, the levels were lower than with the 35S promoter, which could have been due to lower gene expression levels. Unexpectedly, however, transcript levels measured by quantitative RT-PCR show that STAPs confer significantly higher expression than the 35S promoter, in particular up to more than 10-fold higher for STAP-5 driving the expression of the *GGPPS2* gene. Several hypotheses can be proposed to account for this negative correlation between transcript levels and product yield. The high expression of the enzymes could lead to negative feedback on the MEP pathway and therefore reduce flux through this pathway. Another possibility is that CBTol is produced in larger amounts with STAPs but is modified by endogenous *N. benthamiana* enzymes (e.g. acyl- or glycosyltransferases, glutathione transferases), as has been shown for mono- and sesquiterpenoids (Ting *et al*., 2013). Here, however, one would have to invoke that the more CBTol that is produced, the more it is modified in proportion. Yet another possibility is that the higher transcript levels are not accompanied by higher translation rates but that on the contrary this leads to reduced translation activity. Testing these different hypotheses will require extensive additional experiments which should be carried out in the future.

Eukaryotic promoters are currently described as containing two major domains, namely the core promoter element, defined as the region where the transcription machinery is recruited and initiates transcription, and the upstream elements, which are bound by TFs that can either activate or repress transcription (Hahn and Young, 2011). It is well established that binding to specific upstream elements by TFs triggers the recruitment of the core transcription machinery and thereby activates transcription (Hahn and Young, 2011). However, hardly anything is known about how the sequence of the core promoter element influences transcriptional activity (Lubliner *et al*., 2013). In an early study, the analysis of 95 yeast promoters led to the identification of a so-called ‘locator’ signal 30–10 bp upstream of the transcription start site (TSS), which is rich in Ts and poor in As, and is ‘stronger’ in promoters with high activity (Maicas and Friesen, 1990). More recently, a survey of 859 yeast core promoters identified T-richness upstream of the TSS as a strong predictor of promoter activity, regardless of whether the promoters are constitutive or inducible (Lubliner *et al*., 2013). As far as we could tell from the available literature, nothing is known about the situation in plants. Because of their short sequence and the presence of an identical DNA-binding site, STAPs constitute an appropriate material for studying the influence of the base composition of the core promoter on transcription activity. However, the identification of significant correlations between expression level and sequence content in our dataset would require a larger number of sequences.

Transcript levels were measured by quantitative RT-PCR for three of the promoters (STAPs 1, 3 and 5; Figure5). The STAP-1 and STAP-3 promoters showed transcript levels that were similar to those of the 35S promoter, a result which is consistent with the GUS expression assays. Surprisingly the STAP-5 promoter showed transcript levels around 10 times higher than the 35S promoter, although GUS expression levels were similar to those with the 35S promoter. The base content between the TSS and the translation initiation codon [i.e. the 5′-untranslated (UTR) region] is known to have a dramatic impact on translation efficiency leading to differences of up to 100-fold in translation efficiency (Rojas-Duran and Gilbert, 2012). It is therefore quite possible that in the case of STAP-5 the high transcript levels do not result in correspondingly high protein expression due to an unfavourable 5′-UTR sequence. One way to circumvent this issue would be to introduce a 5′-UTR sequence known to enhance translation efficiency, such as the Ω–translation enhancer of the tobacco mosaic virus (Sleat *et al*., 1987).

Our design for the STAPs was based on reports that identified the presence of AvrBs3-binding sites within 100 bp of the transcription start site of genes activated by AvrBs3, a TAL-effector from the plant pathogenic bacterium *Xanthomonas campestris* (Kay *et al*., 2007, 2009; Romer *et al*., 2007). Furthermore, Kay and collaborators showed that a TATA box immediately downstream of the TALE-binding site leads to greatly increased expression (Kay *et al*., 2009). Although our design gave satisfactory results, further variations could provide additional regulatory options. For example, adding multiple TALE-binding sites could lead to increased expression, as shown in mammalian cells (Perez-Pinera *et al*., 2013). However, since the strongest STAPs are almost on a par with the viral 35S promoter this may be of limited interest. More relevant would be the insertion of TALE-binding sites for positive or negative feedback regulation. Positive feedback can simply be achieved by adding a DNA-binding site for another dTALE in the promoter driving the first dTALE. Negative feedback can be achieved in the same way but by replacing the activation domain of the second TALE by a repressor domain. Transforming dTALEs into transcriptional repressors has been successfully carried out in various eukaryotic organisms including plants (Blount *et al*., 2012; Mahfouz *et al*., 2012). Thus, our STAPs provide the starting material for designing complex regulatory circuits that could include transcriptional cascades, Boolean operators, as well as positive and negative feedback loops.

Given the ever-increasing number of genome sequences becoming available, including from higher plants, it will be possible to design TALE DNA-binding sequences that do not, or only minimally, interfere with endogenous gene expression, and conversely that are not bound by endogenous TFs. Thus, the dTALE/STAPs system can be considered a truly orthogonal system that neither depends nor interferes with endogenous transcription activation networks. The relevance of this system for metabolic engineering is obvious. Our transient expression assay for diterpene production in *N. benthamiana* demonstrated that several genes can be co-expressed by the dTALE/STAPs system at levels that are comparable to the strong CaMV 35S promoter. While using the same promoter to co-express multiple genes in a transient assay does work properly, in stable transgenic plants this can lead to undesirable consequences such as gene silencing (Peremarti *et al*., 2010). Therefore, a particularly relevant application of these promoters will be in stable transgenic plants, where coordinated tissue-specific expression of pathway genes can be tested without requiring the identification of multiple endogenous promoters conferring the same expression profile and with different expression levels. Hence, by simply changing the promoter driving the expression of the activating dTALE and keeping the rest of the construct identical, it will be possible with minimum cloning effort to test different tissues in parallel for their performance in producing a compound of interest. For example, one could compare tissues such as the seed endosperm, the epidermis, glandular trichomes or root hairs, for which specific promoters are available (Potenza *et al*., 2004; Tissier, 2012).

Recently, the discovery of the bacterial immune system based on the CRISPR/Cas9 system has opened novel opportunities for genome engineering and synthetic biology (Fineran and Dy, 2014; Hsu *et al*., 2014; van der Oost *et al*., 2014). The CRISPR/Cas9 enzyme is a nuclease whose sequence specificity is determined by a guide RNA (gRNA) containing the target sequence (van der Oost *et al*., 2014). As such, CRISPR/Cas9 is perfectly suited for genome editing, with the possibility of simultaneously targeting several loci by co-expressing or even concatenating multiple gRNAs, which can be easily cloned due to their small size (Hsu *et al*., 2014). In this regard, CRISPR/Cas9 genome editing is simpler to implement than TALE-based editing, because for each locus to be targeted, two new dTALEs need to be designed and expressed (de Lange *et al*., 2014). The CRISPR/Cas system was also modified to generate TFs (CRISPR/Cas-TF), whose specificity is determined by gRNAs (Nissim *et al*., 2014). Typically, gRNAs are expressed from RNA pol III promoters which are constitutive, and therefore provide no flexibility for tissue- or condition-specific expression. One solution to this problem is to express the gRNAs by RNA pol II promoters and edit the RNA with the Csy4 enzyme to ensure processing of the gRNA in the nucleus (Qi *et al*., 2012), but the functionality of Csy4 remains to be shown in plants. In addition to this added complexity, the fact that the specificity of the CRISPR/Cas-TF is determined at the RNA level, and not at the protein level as with the TALEs, constitutes a limitation for the design of more complex regulatory networks with activating or repressing steps. Because all CRISPR/Cas proteins will recognize the gRNAs and their target regardless of whether they are modified to be transcriptional activators or repressors, co-expression of both types of CRISPR/Cas-TFs in a single cell would result in unpredictable expression of the target genes. Also, a single CRISPR/Cas-TF protein controlling the whole network cannot act simultaneously as a repressor and activator on different targets. Thus, to introduce a negative regulatory step in a CRISPR/Cas regulatory network, Nissim *et al*. (2014) had recourse to a miRNA. In contrast, distinct dTALEs with either repressor or activating properties can be co-expressed, thus allowing much more flexibility and possibilities for the design of regulatory networks (de Lange *et al*., 2014).

These STAPs can be used for metabolic engineering of complex biosynthetic pathways requiring the simultaneous and tunable expression of multiple genes. This is particularly relevant in plants, where specific tissues or conditions can then be tested and compared for their performance in producing specific classes of compounds. Furthermore, these STAPs provide the basis for the design and construction of complex regulatory networks that contain transcriptional cascades and positive and negative feedback loops. Thus, their potential reaches beyond the field of metabolic engineering.

## Experimental procedures

### Construction of a STAPs library

To generate a library of promoter sequences as described in Figure1, two degenerate primers, Talpro7 and Talpro8 (see sequences below), containing the TALE DNA-binding site (effector binding element 2 or EBE-002, described in Weber *et al*. (2011) as the binding site for dTALE-2) and degenerate sequences on either side of the DNA-binding site were made and annealed at a concentration of 0.2 μm each in a 50 μl reaction mix with the KOD polymerase (Merck Millipore, https://www.merckmillipore.com). Primer extension was carried out by first denaturing for 5 min at 94°C, then annealing at 60°C for 30 sec and extending at 72°C for 30 sec. The product was then purified on a Qiaquick column (Qiagen, http://www.qiagen.com/) and inserted into vector pAGM1311 (universal level –1 cloning vector) (Engler *et al*., 2014) using a standard Golden Gate cloning procedure with *Bsa*I and T4 DNA ligase (Werner *et al*., 2012). An aliquot (100 μl) of the transformed bacteria was spread over two Petri dishes for evaluation of the complexity of the library, and the remainder of the transformation inoculated in liquid culture for library preparation. Then DNA from the whole library was prepared and further subcloned into the level 0 vector pICH41295 (Engler *et al*., 2014) using *Bpi*I and T4 DNA ligase. The strategy of cloning the library in two steps (level −1 and 0) results in a higher amount of promoters with a correct structure compatible with Golden Gate cloning. In particular, the two successive cloning steps that use *Bsa*I and then *Bpi*I for cloning result in the elimination of promoters that may have contained *Bpi*I or *Bsa*I sites in internal random sequences. The resulting level 0 constructs (pAGT582-n) were then picked individually, checked for the presence of single promoter sequences after digestion with *Bsa*I, sequenced and used for further cloning. The sequences of the 43 promoters are provided in Data S1. Talpro7: 5′-ttt ggtctc a acat GGAG (*n*)_19_ tccccgcatagctgaacatc. Talpro8: 5′-ttt ggtctc a acaa CATT (*n*)_43_ ttatata g atgttcagctatgcgggg (the capital letters indicate sequences that will become part of the *Bsa*I cleavage sites in the final level 0 modules).

### Reporter constructs

The STAPs coming from the pAGT582-*n* vectors were cloned either in front of a *GUS* reporter gene (plasmid pICH75111) (Engler *et al*., 2014) and the ocs terminator (pICH41432) (Engler *et al*., 2014) to give pAGT615-*n* vectors, or in front of the *GFP* reporter gene (pICH4153) (Engler *et al*., 2014) and the ocs terminator to give the pAGT917-*n* vectors. The constructs were made in the T-DNA vector pICH75044 (Engler *et al*., 2014). All clonings were made using standard Golden Gate cloning procedure with the appropriate restriction enzymes (Engler *et al*., 2008).

### GUS transient assays

All T-DNA vectors were transformed into *A. tumefaciens* strain GV3101 (Koncz and Schell, 1986). *Nicotiana benthamiana* plants were grown in a greenhouse maintained at a constant temperature of 22°C with 82% humidity. For the GUS assays, the pAGT615-*n* constructs were co-infiltrated with the TALE containing the DNA-binding domain specific for the EBE002 sequence (construct pICH74043; Weber *et al*., 2011) and a 35S:*GFP* construct as internal reference (construct pAGM4731; Engler *et al*., 2014). As a control, the pAGT615-*n* constructs were also infiltrated without the TALE. A construct with 35S:*GUS* (pICH75181; Engler *et al*., 2014) co-infiltrated with 35S:*GFP* was used as a positive control and external reference. We infiltrated the reporter constructs with and without the TALE and a positive control (35S:*GUS*) on different sectors of the same leaf to eliminate leaf-to-leaf variation. *Agrobacterium* strains were grown in Luria–Bertani medium, resuspended in infiltration medium [10 mm 2-(*N*-morpholine)-ethansulphonic acid (MES), 10 mm MgSO_4_] at a final OD_600_ of 0.3 for infiltration. The plants were then replaced in the greenhouse for 5 days before analysis. For quantitative GUS assays, leaf discs of diameter 0.9 cm within the infiltrated areas were excised and transferred to microtubes containing three 0.2-mm steel beads and immediately frozen in liquid N_2_. Frozen leaf tissues were then homogenized twice using a Retsch mixer mill MM400 at a frequency of 30 Hz for 1 min. The leaf powder was then resuspended in 1 ml phosphate buffered saline (pH 7.4), vortexed and filtered through a 0.2 μm polytetrafluoroethylene filter on a 96-well plate (Chromafil, Macherey-Nagel, http://www.mn-net.com/). The GUS activity was measured with the fluorogenic 4-methylumbelliferyl β-d-glucopyranoside (MUG) substrate using published procedures at 10, 30 and 60 min (Jefferson *et al*., 1987). In parallel, 200 μl of the extracts were used to measure GFP fluorescence. The GFP fluorescence of each sample was used as the normalization factor. The strength of the individual promoters was then expressed as a percentage of the expression with the 35S promoter, based on the control from the same leaf.

### GFP transient assays

Selected promoters from the pAGT582-*n* series were cloned into a level 1 vector (pICH47732) in front of *GFP* (vector pICH41531; Engler *et al*., 2014) and the ocs terminator (pICH41432). The constructs were introduced into *Agrobacterium* GV3101 and expressed in *N. benthamiana* with or without the TALE-EBE002 (see above). The 35S:*GFP* construct (pAGM4731, see above) was used as a positive control. The plants were incubated for 3–5 days and photos were taken under a UV lamp.

### Diterpene metabolic engineering constructs

The coding sequences of the *SlDXS2* (pAGT345), *NtGGPPS2* (pAGT169) and *CBTS2a* (pAGT238) genes were those described in Brückner and Tissier (2013). They were cloned behind the promoters from pAGT582-3, pAGT582-5 and pAGT582-1, respectively. The terminators used were Sl vATPase1 (pICH71431), Ocs (pICH 41432), and CBTS (pAGT168), respectively (Brückner and Tissier, 2013; Engler *et al*., 2014). These fragments were assembled into the level 1 vectors pICH47751, pICH47742 and pICH47802, respectively. The vectors were transformed into *A. tumefaciens* GV3101 and used for *N. benthamiana* leaf infiltration as described above. The corresponding constructs with the 35S promoter were as described in Brückner and Tissier (2013). Metabolite extraction and diterpene GC-MS measurements were performed as described in Brückner and Tissier (2013) with the following modifications. The 1 ml hexane extracts from the six leaf discs per sample were not evaporated and 160 μl of these extracts were combined with 40 μl of internal standard (IS) (50 μm sclareol stock solution in hexane), mixed, centrifuged and transferred to a GC-vial. Samples were measured on a QP2010 Ultra GC-MS system (Shimadzu, http://www.shimadzu.com/) in the following conditions. One microlitre was injected splitless at 250°C with a 1-min sampling time in a RXi-5Sil MS 30 m, 0.25-mm diameter column (Restek, http://www.restek.com/). Elution was done with a constant He flow of 0.9 ml min^−1^. The oven temperature was raised from 50°C to 300°C (7°C min^−1^), then to 320°C (50°C min^−1^) and held for 2 min. The interface and the ion source temperatures were 300°C and 250°C, respectively, and the ionization voltage was set at 70 V.

### Quantitative gene expression analysis in infiltrated leaf tissue

Total RNA was extracted from infiltrated leaf discs from which diterpenes were extracted by a prior hexane wash. Leaf discs were homogenized to a fine powder using a Mixer Mill MM-400 (Retsch, http://www.retsch.com/) and RNA was extracted with the Spectrum™ Plant Total RNA Kit (Sigma-Aldrich, http://www.sigma-aldrich.com/) according to the manufacturer’s specifications. Any genomic contamination was removed before cDNA synthesis using the DNA-free Kit (Ambion, http://www.lifetechnologies.com/uk/en/home/brands/ambion.html) according to the manufacturer’s instructions. The first strand of cDNA was synthesized from 1 μg total RNA using a Maxima H Minus First Strand cDNA Synthesis Kit (Thermo Scientific, http://www.thermoscientific.com/) and a mix of oligo(dT)18 and random hexamer primers. The cDNA was diluted 10-fold and 1 μl was used in a 10-μl reaction mix containing 1 μl (2 pmol μl^−1^) of both forward and reverse primers and 2 μl my-Budget 5 ×  EvaGreen QPCR Mix II (Bio&Sell, http://www.bio-sell.de/). Quantitative real-time PCR was performed in 96-well plates using the CFX Connect real-time PCR detection system (Bio-Rad, http://www.bio-rad.com/). The thermal profile of the reaction was as follows: an initial denaturation at 95°C for 15 min, followed by 40 cycles at 95°C for 15 sec and 58°C for 30 sec with fluorescence acquisition after each cycle. To verify primer specificity, a dissociation curve was finally generated by increasing the temperature from 65 to 95°C. Quantitative (q)PCR primers for the amplification of *CBTS2a*,*DXS2* and *GGPPS2* genes were designed using the Primer3 program (Untergrasser *et al*., 2012). The *N. benthamiana* eukaryotic elongation factor 1a was selected as the endogenous reference for normalization of transcript levels and PCR amplified using primers as described in Liu *et al*. (2012). To check the specificity of the *CBTS2a*,*DXS2* and *GGPPS2* primers, qPCR was also performed on *N. benthamiana* cDNA and no amplification could be detected in any of the control samples (Figure5). The sequences of the oligonucleotides used for real-time PCR are provided in Data S2. All the cDNA samples were amplified in three technical replicates from the same RNA preparation and the mean value was considered. Expression of transgenes in infiltrated leaf tissue was calculated using the 2^(ΔCt)^ method (Livak and Schmittgen, 2001).

## Supporting Information

**Figure S1.** Correlation between gene expression and cembratrienol (CBTol) levels.

**Data S1.**Sequences of the 43 synthetic transcription activator-like effector-activated promoters (STAPs). **Data S2.** Sequences of the primers used for quantitative gene expression analysis.
