# Supplementary material for: A library of synthetic transcription activator-like effector-activated promoters for coordinated orthogonal gene expression in plants
Source: Plant J. 2015 Apr 29;82(4):707–16. doi: 10.1111/tpj.12843 (PMC4691316; doi:10.1111/tpj.12843)
Supplement: Supplementary file 1 [file tpj0082-0707-sd1.pdf]

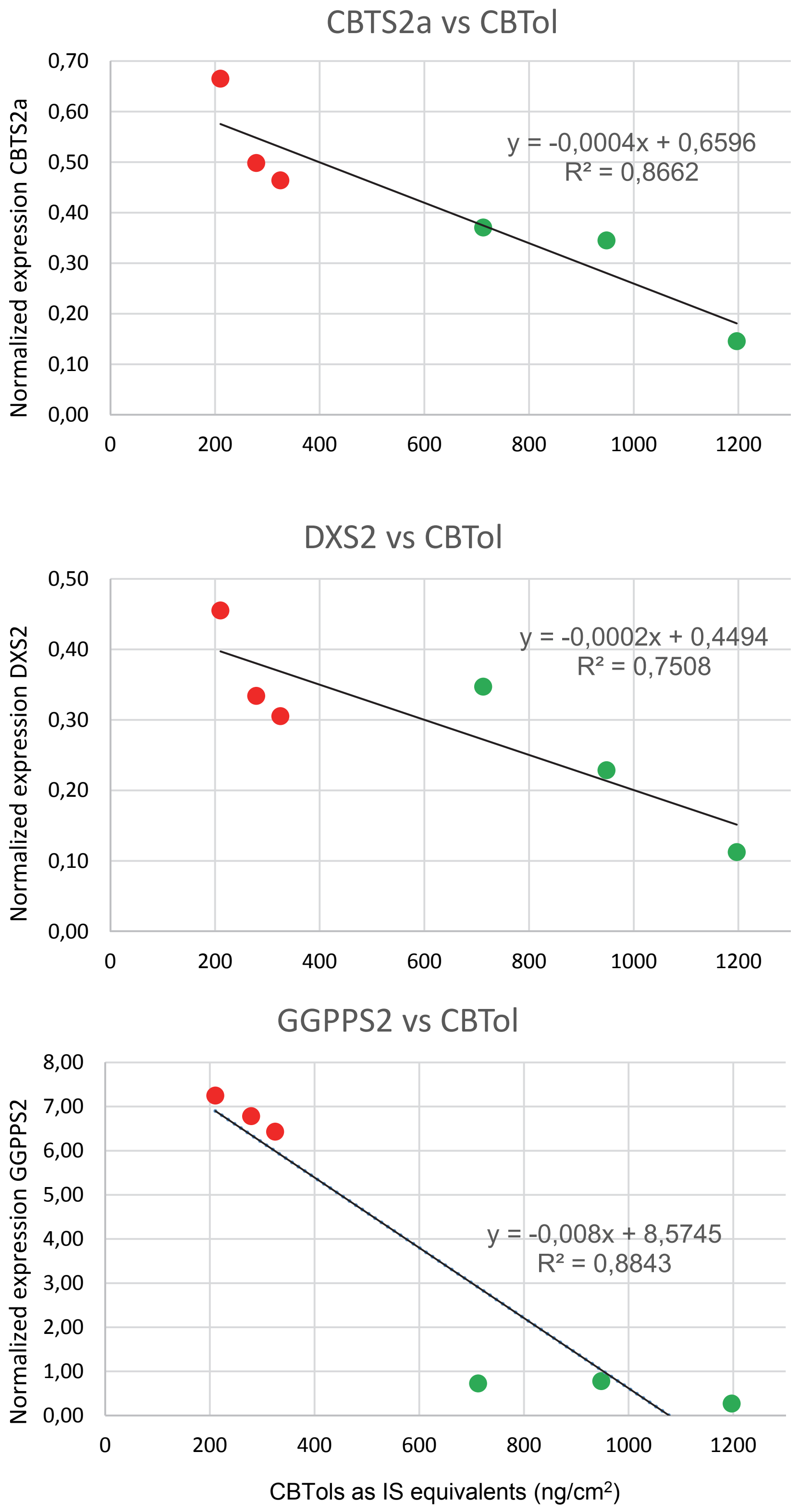

Supplemental Figure 1. Correlation between gene expression and CBTol levels.
Expression data from Figure 5 was plotted against CBTol levels. Trend lines and their corresponding parameters are shown. Red and green dots correspond to samples with the STAPs and 35S promoter respectively.
