# Supplementary material for: A library of synthetic transcription activator-like effector-activated promoters for coordinated orthogonal gene expression in plants
Source: Plant J. 2015 Apr 29;82(4):707–16. doi: 10.1111/tpj.12843 (PMC4691316; doi:10.1111/tpj.12843)
Supplement: Supplementary file 2 [file tpj0082-0707-sd2.pdf]

Supplemental data

1. **Sequences of the STAPs**

The 19 base long DNA binding site of the TALE is indicated in bold letters

>PAGT582_1
GGAGGTTATCACACTTGGTAATT**TCCCCGCATAGCTGAACAT**CTATACAACACTCATCG
CGCGGAGAAACGCGCAACAAATTGGAGGCGCATTAATG

>PAGT582_2
GGAGCTTACGTAGCAGCACGGCC**TCCCCGCATAGCTGAACAT**CTATATAAATCTTAGCC
CGACTGCACCGTGAGGAAAAGAGGGTTTATATATAATG

>PAGT582_3
GGAGGTCGTCAAATGATAAGTAC**TCCCCGCATAGCTGAACAT**CTATATAACGCAGCAAA
TGGTTGAAACGTATTAGCAACAGCTAAACCTTCGAATGT

>PAGT582_4
GGAGGCGTGTCGTTTTAGTGAGG**TCCCCGCATAGCTGAACAT**CTATATAAGGTTTTGCT
ATTCATTGAAAGCAGTAGTGACTGATTTGTATATAATG

>PAGT582_5
GGAGGTTCTGTGGGTTTTTTGGG**TCCCCGCATAGCTGAACAT**CTATATAATGAGAAACA
GGGGCAAATCCCCGAAAACAAATAACGCGAAGCAATG

>PAGT582_7
GGAGATGTGCCATGGTCGGGGGC**TCCCCGCATAGCTGAACAT**CTATATAATCGGAATG
AATTGCAAATAGGGATGTTATAGGGTTATCTCGGCAATG

>PAGT582_8
GGAGAGATGATTGTTCGTAATAT**TCCCCGCATAGCTGAACAT**CTATATAAGAGACGAGC
TACCACGATAAGCGCAGCAAAAGACACATACGTTAATG

>PAGT582_9
GGAGGTGCCTTTTTAAAAGGGTT**TCCCCGCATAGCTGAACAT**CTATATAAGAAACACGG
ACGCCGAAACCCCCCGCCTAGTTGACTCTCCGGAATG

>PAGT582_10
GAGTGTGAACGTGGTGATGTGG**TCCCCGCATAGCTGAACAT**CTATATAAAAACTGTAAC
AGAACTGCTGCTCAACTAGTCTCCAACTATATAAATG

>PAGT582_13
GGAGGTAAGTTCTGGCCTCTGGG**TCCCCGCATAGCTGAACAT**CTATATAATAGGACAG
TAAGATATGCCAGACAATGTAGCACGTAGAACGGAAATG

>PAGT582_14
GGAGCACCTTGGAAAACAGGTGG**TCCCCGCATAGCTGAACAT**CTATATAAGAAATTAGT
AACTACGCTTTCAAAGACATCTTAATTAATTAGAATG

>PAGT582_15
GGAGAGTGGGCGCGTTTGGTAAG**TCCCCGCATAGCTGAACAT**CTATATAAATAAGCTG
CCTACGATTTAGCGCGACACAATTCGACGGTTGGAAATG

>PAGT582_21
GGAGGCCGTCGGGGGGGGGTCTG**TCCCCGCATAGCTGAACAT**CTATATAATACCGGTT
ATCCAGCGACAACCGTCAATAACTTAACATTGAACAATG

>PAGT582_23
GGAGTTAAGTAGGCTGTTCTTAT**TCCCCGCATAGCTGAACAT**CTATATAAATAGCTCAGT
TGCCTTAAACGTAGACAGTCTATTACACTAATAAATG

>PAGT582_24
GGAGGTTTATACTTTCTCTAATT**TCCCCGCATAGCTGAACAT**CTATATAAACTGGAACTT
TCACTGACCTGACAGTGCTTTCATTGACGCGCGAATG

>PAGT582_25
GGAGAAGCTCACCTGCATCCTTG**TCCCCGCATAGCTGAACAT**CTATATAAAATTTATAC
ATTTAAACCAAACCCTATAAAATTTCTGTCCGCGAATG

>PAGT582_29
GGAGTAATGTACAGCTTCGCGGC**TCCCCGCATAGCTGAACAT**CTATATAATAACTGTTC
TACATACTCTCGGATAAATAACGCAACTTAATATAATG

>PAGT582_30
GGAGTCCCGGATCCTGGTTTCAC**TCCCCGCATAGCTGAACAT**CTATATAAAACAAATCT
CGTCAAGCAAACCAATTACGATGGTCCGGGAAGGAATG

>PAGT582_34
GGAGGGGTGGGGGGGTTACCGTC**TCCCCGCATAGCTGAACAT**CTATATAAAACCAATC
GACGACTCATTGGAATGCGTCCTGCTCCAAATAGCAATG

>PAGT582_36
GGAGCTGCAATGCTTACTGTTAT**TCCCCGCATAGCTGAACAT**CTATATAAAGGTTGAGA
AGTGGCACTTGCAATAATTGCCGGGCCCATGGGAAATG

>PAGT582_37
GGAGAGAGGTGGGTCGCGGGCAT**TCCCCGCATAGCTGAACAT**CTATATAAAAAGCAGT
TCATACGGGAACGCAGACTAGTCACGAAATTCCGCAATG

>PAGT582_38
GGAGGCGCGTTTGATTGGGGGGG**TCCCCGCATAGCTGAACAT**CTATATAAAACTGATA
ACTTAGTCAGACTAGCAAATGCTCACAATACAAAGAATG

>PAGT582_39
GGAGGAACCGCGGTTCGGGAGCG**TCCCCGCATAGCTGAACAT**CTATATAACAGGGGC
GGGATATGATATAGAAACACCAGGGTTAGTAACAAGAATG

>PAGT582_40
GGAGCAATGGCAGAGGGGTATGG**TCCCCGCATAGCTGAACAT**CTATATAAAATTGGGA
CCTATGATAATGCTGTAGAACCTCATAAATGATGGAATG

>PAGT582_41
GGAGGGTTTGTTACCGTGTATTC**TCCCCGCATAGCTGAACAT**CTATATAACGAGCGGTC
GAAAGCTTCAAAATTCAATGAGTAAGACCCCAACAATG

>PAGT582_42
GGAGTCTTGCCCATACTCAGTTC**TCCCCGCATAGCTGAACAT**CTATATAATGTCTCTCG
AATGACACCGATACTTCGATTTAGCAGGCTGCCCAATG

>PAGT582_43
GGAGGCATTACGGGCGTAGTTTG**TCCCCGCATAGCTGAACAT**CTATATAATCGACCAG
AGCAACGTTCTCGCCTAGGCGCGCCTTACCGGGAAAATG

>PAGT582_44
GGAGGTATCCCGAGGGCGAATGG**TCCCCGCATAGCTGAACAT**CTATATAAGGCATCTG
GTTAGCAAGCCTCACCAGCCGTGAACAATATAATCAATG

>PAGT582_45
GGAGTCAGAGTGTGGTCGTGTTT**TCCCCGCATAGCTGAACAT**CTATATAAGTTTACACC
TGCCCGACCTATTCCTTGATGAGCTCGCGCACCGAATG

>PAGT582_47
GGAGCACTTAGGTTAACTGCGCG**TCCCCGCATAGCTGAACAT**CTATATAAGGAACAGA
CGGCTAACCCATCAATGGAACAACGAACGGCCCATAATG

>PAGT582_48
GGAGTGGTTTTAGCACAGACAAC**TCCCCGCATAGCTGAACAT**CTATATAAGGAACCGAC
TCTCTCCCGCCCCCGACACAGGACCCCCTCTGGTAATG

>PAGT582_52
GGAGCATGCTGGGGTTGGAGGGG**TCCCCGCATAGCTGAACAT**CTATATAACCCGGCC
CCTCAATTAACCCTGCTCAAAGCCAAAGCAACAATCAATG

>PAGT582_53
GGAGTGGGCTGAGGGGGACGGGT**TCCCCGCATAGCTGAACAT**CTATATAACCCAACGT
GAGATCACGTTTACAATACTCGTCTTGACTGACACAATG

>PAGT582_54
GGAGGGCATGAGGTTAGCTAGTT**TCCCCGCATAGCTGAACAT**CTATATAATGAATGTGT
GAGAAGCTTTGACAAGCTTCGCCCAGCGGCCCTAAATGT

>PAGT582_56
GGAGATATTCCTCGGCTGTCACT**TCCCCGCATAGCTGAACAT**CTATATAATCAAATTCCT
AGGGGCTGCCATGGTTACCCCTTGCTATGTAGCAATG

>PAGT582_58
GAGTGCGGGGAGTTGGTATGGG**TCCCTGCATAGCTGAACAT**CTATATAAATAACGGCC
CTGAGTGTCGCTAGCAAATGCACCAAACCAACTCAATG
>PAGT582_60
GGAGCGATATGTTTAATCGGCAT**TCCCCGCATAGCTGAACAT**CTATATAAATCCGCGCC
TTAAATACGGTTGTTTGCCTATTTTGAGCGAGTAAATG

>PAGT582_62
GGAGTATTGGTCCACTTGCTCCG**TCCCCGCATAGCTGAACAT**CTATATAACGCAGAAAC
CTCGCAACTGACGTTCCGATTGCGATAAACACACAATG

>PAGT582_65
GGAGTTGTAAACTCCAGGTTAGA**TCCCCGCATAGCTGAACAT**CTATATAAATCGCCCAT
TGCCCAGGACACGCAACCCTGCAACAATCAGTACAATG

>PAGT582_69
GGAGCCCTTGCTATGACGATGCC**TCCCCGCATAGCTGAACAT**CTATATAAAAATTCCAT
AACGTACAGAACGAAGAACGGACCCATATACAATAATG

>PAGT582_73
GGAGGCGCGGGCAGGGGTTGGGT**TCCCCGCATAGCTGAACAT**CTATATAATCACCCAA
CGCCGCATGCAACAACTAGTAACAAGGCCATTGAGAATG

>PAGT582_78
GGAGCTTTTTACGGGGCACCTGT**TCCCCGCATAGCTGAACAT**CTATATAACAAAATAAC
AGATTGTCTGTCCAAGAGGCAGACATTCGTTCATAATG

2. Sequences of the primers used for quantitative gene expression analysis

NsCBTS2a-1106F, 5’- CGATCCAAAGATGGGACGA-3’,
NsCBTS2a-1257R, 5’- CATCTCTTTTTTCGCATAGTAGATGT-3’,
NtGGPPS2-85F, 5’- CCTCCACAGAGACATTCTTATAGTTTC-3’,
NtGGPPS2-227R, 5’- TCAAAGTCAAACTTAGGCAAGATG-3’,
SlDXS2-556F, 5’- AGCATCTCCGCTGGTCTT-3’,
SlDXS2-699R, 5’- CGAGTCAAGGAATCCTGCAT-3’.
