# Supplementary material for: A library of synthetic transcription activator-like effector-activated promoters for coordinated orthogonal gene expression in plants
Source: Plant J. 2015 Apr 29;82(4):707–16. doi: 10.1111/tpj.12843 (PMC4691316; doi:10.1111/tpj.12843)
Supplement: Supplementary file 3 [file tpj0082-0707-sd3.docx]

**Full Legend of supporting information**

**Supplemental data 1. Sequences of the fourty-three STAPs.** The 19 base long DNA binding site of the TALE is indicated in bold letters.

**Supplemental data 2. Sequences of the primers used for quantitative gene expression analysis**

**Supplemental Figure.1 Correlation between gene expression and CBTol levels**

Expression data from Figure 5 was plotted against CBTol levels. Trend lines and their corresponding parameters are shown. Red and green dots correspond to samples with the STAPs and 35S promoter respectively.
